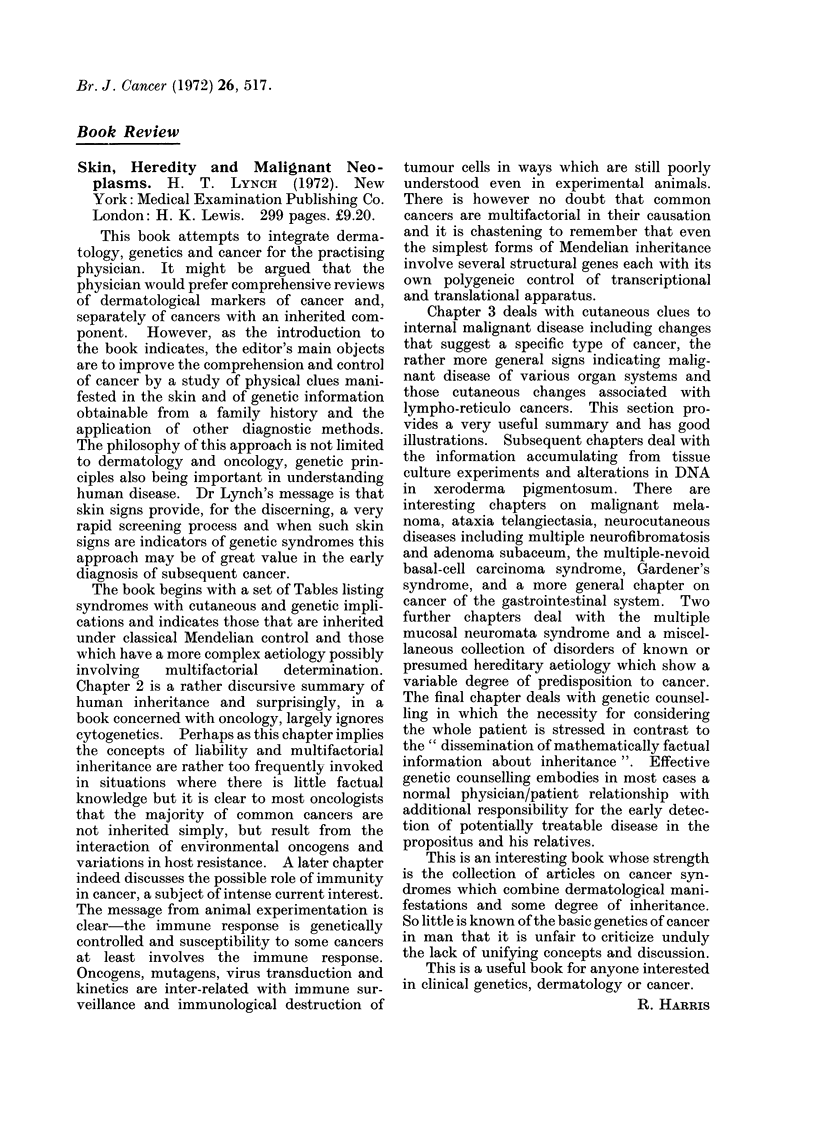# Skin, Heredity and Malignant Neoplasms

**Published:** 1972-12

**Authors:** R. Harris


*Br. J. Cancer* (1972) **26**, 517.

### *Book Review*

**Skin, Heredity and Malignant Neoplasms.** H. T. LYNCH (1972). New York: Medical Examination Publishing Co. London: H. K. Lewis. 299 pages. £9.20.

This book attempts to integrate dermatology, genetics and cancer for the practising physician. It might be argued that the physician would prefer comprehensive reviews of dermatological markers of cancer and, separately of cancers with an inherited component. However, as the introduction to the book indicates, the editor's main objects are to improve the comprehension and control of cancer by a study of physical clues manifested in the skin and of genetic information obtainable from a family history and the application of other diagnostic methods. The philosophy of this approach is not limited to dermatology and oncology, genetic principles also being important in understanding human disease. Dr Lynch's message is that skin signs provide, for the discerning, a very rapid screening process and when such skin signs are indicators of genetic syndromes this approach may be of great value in the early diagnosis of subsequent cancer.

The book begins with a set of Tables listing syndromes with cutaneous and genetic implications and indicates those that are inherited under classical Mendelian control and those which have a more complex aetiology possibly involving multifactorial determination. Chapter 2 is a rather discursive summary of human inheritance and surprisingly, in a book concerned with oncology, largely ignores cytogenetics. Perhaps as this chapter implies the concepts of liability and multifactorial inheritance are rather too frequently invoked in situations where there is little factual knowledge but it is clear to most oncologists that the majority of common cancers are not inherited simply, but result from the interaction of environmental oncogens and variations in host resistance. A later chapter indeed discusses the possible role of immunity in cancer, a subject of intense current interest. The message from animal experimentation is clear—the immune response is genetically controlled and susceptibility to some cancers at least involves the immune response. Oncogens, mutagens, virus transduction and kinetics are inter-related with immune surveillance and immunological destruction of tumour cells in ways which are still poorly understood even in experimental animals. There is however no doubt that common cancers are multifactorial in their causation and it is chastening to remember that even the simplest forms of Mendelian inheritance involve several structural genes each with its own polygeneic control of transcriptional and translational apparatus.

Chapter 3 deals with cutaneous clues to internal malignant disease including changes that suggest a specific type of cancer, the rather more general signs indicating malignant disease of various organ systems and those cutaneous changes associated with lympho-reticulo cancers. This section provides a very useful summary and has good illustrations. Subsequent chapters deal with the information accumulating from tissue culture experiments and alterations in DNA in xeroderma pigmentosum. There are interesting chapters on malignant melanoma, ataxia telangiectasia, neurocutaneous diseases including multiple neurofibromatosis and adenoma subaceum, the multiple-nevoid basal-cell carcinoma syndrome, Gardener's syndrome, and a more general chapter on cancer of the gastrointestinal system. Two further chapters deal with the multiple mucosal neuromata syndrome and a miscellaneous collection of disorders of known or presumed hereditary aetiology which show a variable degree of predisposition to cancer. The final chapter deals with genetic counselling in which the necessity for considering the whole patient is stressed in contrast to the " dissemination of mathematically factual information about inheritance ". Effective genetic counselling embodies in most cases a normal physician/patient relationship with additional responsibility for the early detection of potentially treatable disease in the propositus and his relatives.

This is an interesting book whose strength is the collection of articles on cancer syndromes which combine dermatological manifestations and some degree of inheritance. So little is known of the basic genetics of cancer in man that it is unfair to criticize unduly the lack of unifying concepts and discussion.

This is a useful book for anyone interested in clinical genetics, dermatology or cancer.

R. HARRIS